# Impact of perioperative clonidine on postoperative respiratory adverse events in children: a post-hoc observational analysis of prospectively collected datasets

**DOI:** 10.1016/j.bjane.2026.844792

**Published:** 2026-07-08

**Authors:** David Sommerfield, Bojana Stepanovic, Daisy Evans, Andy Jeon, Anne Louise de Barros Garioud, Aine Sommerfield, Neil Hauser, R. Nazim Khan, Britta S. von Ungern-Sternberg

**Affiliations:** a Perth Children’s Hospital, Child and Adolescent Health Service, Department of Anaesthesia and Pain Medicine, Perth, Australia; b The University of Western Australia, Medical School, Perth, Australia; c The University of Western Australia, Institute for Paediatric Perioperative Excellence, Perth, Australia; d The Kids Research Institute Australia, Perioperative Medicine Team, Perth, Australia; e The University of Western Australia, School of Physics, Mathematics and Computing, Perth, Australia; f Copenhagen University Hospital, Department of Anaesthesiology, Juliane Marie Centre, Rigshospitalet, Copenhagen, Denmark; g University of Copenhagen, Department of Clinical Medicine, Copenhagen, Denmark; h The University of Western Australia, Department of Mathematics and Statistics, Perth, Australia

**Keywords:** Clonidine, Patient Outcome Assessment, Pediatric Anesthesia, Perioperative Period, Postoperative Complications, Signs and Symptoms, Respiratory

## Abstract

**Background:**

Clonidine, an alpha-2-agonist, has several applications in pediatric anesthesia. Sympathetic nervous system-mediated pathways contribute to airway reactivity, suggesting that clonidine could mitigate respiratory adverse events similarly to more selective agents.

**Methods:**

This was a post-hoc analysis of prospectively collected observational datasets from three research projects on children (1‒16 years) undergoing elective extracapsular (adeno-) tonsillectomies between January 2018 and November 2021. Patients’ study data sheets and medical records were reviewed for clonidine use. The primary outcome was postoperative respiratory events (at emergence from anesthesia or in the post-anesthesia care unit). Post-anesthesia care unit length of stay was also monitored.

**Results:**

The cohort consisted of 512 patients. The tonsillectomies were performed using coblation or electrocautery, local anesthetic infiltration; the airway was managed with a flexible supraglottic airway (97%). In the unadjusted cohort, postoperative respiratory events occurred in 44/306 children receiving clonidine (14%) and 50/203 children not receiving clonidine (25%) (OR = 0.616; 95% CI 0.361–1.047; p = 0.074). After propensity score matching, the between-group difference narrowed to 5 percentage points in the complete-case dataset and 4 percentage points in the multiply imputed dataset, with no statistically significant association between clonidine and postoperative respiratory events. Clinically, clonidine use did not prolong stay in PACU, 48.2 minutes (±22.5), compared with 50.0 minutes (±23.5).

**Conclusions:**

This post-hoc analysis found no statistical reduction in postoperative respiratory events associated with perioperative clonidine in pediatric patients following extracapsular (adeno)tonsillectomy and a flexible supraglottic airway. Clonidine use did not prolong time in PACU.

## Introduction

Clonidine is a centrally acting alpha-2 agonist and a popular agent in pediatric anesthesia with several applications, including premedication, prevention of emergence delirium, antiemesis, and as an analgesic adjunct, both systemically and in regional anesthesia.^1^ Intraoperatively, clonidine reduces sympathetic outflow and attenuates the neurohormonal response to surgical stress.^2^ Some evidence suggests that anxiety and sympathetic nervous system-mediated pathways can contribute to airway reactivity.^3^, ^4^, ^5^ Dexmedetomidine, a more selective alpha-2 agonist, has been beneficial in an animal reactive airway model.^6^ This introduces the possibility that clonidine might similarly mitigate respiratory complications. Two studies have reported a lower rate of respiratory adverse events following intranasal dexmedetomidine.^7^^,^^8^

The 7^th^ National Audit Project of the Royal College of Anaesthetists UK found that severe hypoxemia was the leading cause of cardiac arrest in patients undergoing non-cardiac surgery.^9^ Serious respiratory adverse events are a leading cause of closed claims for anesthesia in the United States.^10^ Perioperative Respiratory Adverse Events (PRAE) occur from the induction of anesthesia until discharge from the Postoperative Care Unit (PACU). The incidence of PRAE is approximately 15%‒30% of pediatric general anesthetics, and it is most frequent in the postoperative phase.^11^, ^12^, ^13^ Tonsillectomy patients are regarded as at a higher risk.^14^

Medications given perioperatively that are active in the early postoperative phase therefore have the potential to reduce adverse respiratory events and improve outcomes. To date, no Randomized Controlled Trials (RCTs) have directly examined the impact of clonidine on adverse respiratory events in children.

The primary aim of this post-hoc analysis of observational datasets was to explore whether the administration of clonidine pre- or intraoperatively reduced the incidence of postoperative respiratory events in children prior to designing a more definitive RCT. A secondary outcome was to assess the effect of clonidine on the time to discharge from the PACU.

## Methods

### Cohort

Post-hoc analysis of prospectively collected observational datasets from three research projects on children undergoing elective (adeno-) tonsillectomies between January 2018 and November 2021: the Bee Pain Free (Child and Adolescent Health Service Human Research Ethics Committee Approval CAHS HREC RGS0000003325, Australia and New Zealand Clinical Trials Registry ACTRN12619001385134),^15^ EMPHASIS (CAHS RGS0000000296; ACTRN12617001379303), and OSATS2 (CAHS RGS0000000014; ACTRN12615001037594)^16^^,^^17^ trials. The Bee Pain Free trial was an interventional study on postoperative pain, while the other two were observational studies investigating PRAE risk factors. Nearly identical perioperative data were collected for each, allowing for a merged analysis. Our report follows Strengthening the Reporting of Observational Studies in Epidemiology (STROBE) guidelines.

Patients between 1 and 16 years old were enrolled from two public and three private centers in Western Australia. The main exclusion criteria were severe cardiopulmonary disease or other significant medical conditions. In all studies, extracapsular tonsillectomy was performed using Coblation^TM^ (Smith Nephew, Watford, UK), BiZact^TM^ (Medtronic PLC, Minneapolis, USA) or bipolar/monopolar electrocautery. Local anesthetic infiltration was usually given by the surgeons, no topical or peri-glottic local anesthetic was used. The indication for surgery in nearly all cases was sleep-disordered breathing. Anesthesia was conducted in accordance with institutional guidelines. Sedative (midazolam or clonidine) premedication was at the discretion of the treating anesthetist, and use was recorded. Induction of general anesthesia was either via incremental inhalation of sevoflurane (up to 8 vol%, with nitrous oxide) or intravenous propofol (> 3 gt; 3 mg.kg^-1^). Sevoflurane was used for anesthesia maintenance in most patients, with a small number receiving a propofol infusion. The airway was managed with a flexible supraglottic airway in the vast majority of children. Deep vs. awake removal of the airway was also recorded.

Analgesia included standardized administration of acetaminophen 15‒20 mg.kg^-1^ orally (preoperatively) or Intravenously (IV) intraoperatively and weight-based parecoxib IV. Opioid use was at the discretion of the treating anesthetist and Oral Morphine Equivalent (OME) was calculated, typically fentanyl 1‒2 μg.kg^-1^, and/or morphine 0.05‒0.1 mg.kg^-1^, pethidine 1 mg.kg^-1^ or hydromorphone 10‒20 mcg.kg^-1^. In a few children, alfentanil or remifentanil was used as part of total intravenous anesthesia maintenance. In the PACU, intravenous fentanyl 0.25 μg.kg^-1^ or morphine 0.25 mg.kg^-1^ was available until tolerating oral medications for acute pain. All patients received dual intraoperative antiemetics.

### Exposure

Clonidine was administered at the discretion of the treating anesthetist. When used, it was typically given as premedication at 2‒4 μg.kg^-1^ or intravenously intraoperatively at 1 μg.kg^-1^. Patients’ study datasheets and medical records were reviewed for the presence or absence of pre- and/or intraoperative clonidine.

### Primary outcome

The primary outcome was postoperative respiratory events, as defined in previous studies on perioperative respiratory adverse events by our group i.e., measured any PRAE recorded at emergence from anesthesia or in the PACU.^12^

• Laryngospasm: complete airway obstruction with associated muscle rigidity of the abdominal and chest walls, leading to the child’s unsuccessful ventilation, unresponsive to simple jaw thrust and CPAP maneuvers.
• Bronchospasm: increased respiratory effort, particularly during expiration and wheeze on auscultation.
• Desaturation: Oxygen saturation < 95% for > 10 secs on pulse oximetry.
• Airway obstruction: the presence of airway obstruction with a snoring noise and/or increased respiratory efforts.
• Severe persistent coughing: pronounced, persistent coughs lasting more than 10 seconds.
• Postoperative stridor: high-pitched sound during breathing in the postoperative period.

Risk factors for PRAE collected in all studies were: history of recurrent wheeze, prior diagnosis or family history of asthma or atopy, Upper Respiratory Tract Infection (URTI) in the past 2 weeks, caregiver smoking, as previously published.^12^^,^^17^

### Statistical analysis

The incidence of postoperative respiratory events was compared for children who received clonidine and those who did not. However, these raw incidences do not account for baseline differences between the two groups. Propensity Score Matching (PSM) was therefore used to adjust for any systematic differences between patients who were and were not given clonidine. Propensity scores were estimated by fitting a logistic regression model for the probability of being given clonidine pre- or intraoperatively. True confounders (variables related to both treatment with clonidine and the outcome of respiratory events) and variables related to the outcome, except treatment with clonidine, were included in the propensity score estimation model,^18^^,^^19^ regardless of their statistical significance level. These included respiratory factors (history of respiratory symptoms, recent upper respiratory tract infection, eczema, family history of asthma and/or eczema, exposure to tobacco smoke, history of wheezing), age, ASA category, type of anesthesia induction, maintenance, airway device, timing of removal of airway device, trial, year of surgery, site (public vs. private), and sex. To address missing data, we used Multiple Imputations by Chained Equations (MICE)^20^ with m = 100 imputations prior to propensity score matching. The log-odds of estimated propensity scores were matched between the clonidine and no-clonidine groups using a 1:1 nearest neighbor approach without replacement, and with a caliper size of 0.2*Standard Deviation (SD). Covariate balance was assessed by consideration of the Standard Mean Difference and Variance Ratio between the clonidine and no-clonidine groups.

To estimate the effect of clonidine on the incidence of postoperative respiratory events, we fitted logistic regression models to the binary outcome occurrence of respiratory events at emergence and/or PACU for both the multiply imputed and complete case propensity-score-matched datasets.^18^^,^^21^ Similarly, to estimate the effect of clonidine on the length of stay in PACU, we fitted a linear model to the continuous outcome of PACU stay duration.

All analyses were performed in R.^22^ Statistical significance was taken at 5% (p < 0.05) unless otherwise stated.

## Results

**Figure 1:**
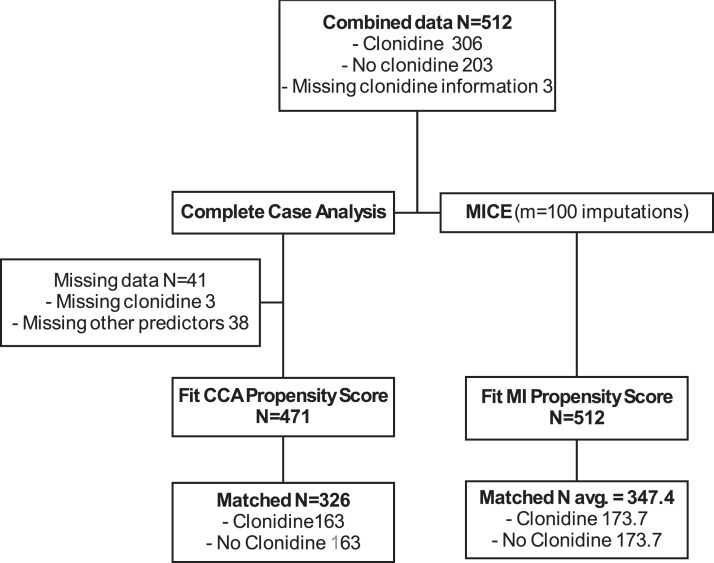
Cohort and propensity score matched datasets. Avg, Average between multiply imputed and matched datasets; CCA, Complete Case Analysis; MI, Multiple Imputation; MICE, Multiple Imputation by Chained Equations.

The cohort consisted of a total of 512 patients enrolled in the three included studies (Fig. 1). Baseline characteristics and anesthesia details grouped by clonidine status are presented in Table 1 and risk factors for adverse respiratory events are presented in Table 2; these are broadly comparable between groups. Fourteen percent (44/306) of the clonidine cohort received it preoperatively but the dose was not recorded on the data sheet, with the remaining patients generally receiving 1 μg.kg^-1^ intraoperatively.

**Table 1:** Baseline and anesthesia characteristics of patients grouped by whether or not they received clonidine pre- or intraoperatively.

Characteristic	No Clonidine (n = 203)	Clonidine (n = 306)
Trial		
BEE	141 (69%)	249 (81%)
EMPHASIS	22 (11%)	35 (11%)
OSATS2	40 (20%)	22 (7%)
Year of Surgery		
2018	14 (6.9%)	18 (5.9%)
2019	36 (18%)	22 (7.2%)
2020	123 (61%)	188 (61%)
2021	30 (15%)	78 (25%)
Site		
Public	185 (91.1%)	198 (65%)
Private	18 (8.9%)	108 (35%)
Age in years, mean (SD)	6.70 (2.97)	6.23 (2.95)
Age range in years		
1 to 5.9	95 (47%)	162 (53%)
6 to 8.9	75 (37%)	99 (32%)
9 and over	33 (16%)	45 (15%)
Weight in KG, median (P25, P75)	22.95 (19.18, 31.60)	21.26 (16.80, 28.25)
Height in CM, mean (SD)	121.9 (17.7)	117.7 (19.9)
BMI z-score for age, mean (SD)	0.54 (1.35)	0.45 (1.49)
BMI Category by Percentile for age		
Underweight (< 5^th^)	11 (6%)	19 (17%)
Healthy Weight (5^th^‒< 85^th^)	119 (60%)	163 (58%)
Overweight (85^th^‒< 95^th^)	22 (11%)	43 (15%)
Obese (95^th^+)	43 (22%)	49 (17%)
Under 2 years old	3 (2%)	8 (3%)
ASA		
I	81 (40%)	135 (44%)
II	120 (59%)	167 (55%)
III	2 (1%)	4 (1%)
Sex: Male / Female	125 (62%) / 78 (38%)	153 (50%) / 153 (50%)
Induction: IV / Inhalation	28 (14%) / 175 (86%)	31 (10%) / 275 (90%)
Maintenance: Sevoflurane / Propofol TIVA	200 (99%) / 3 (1%)	288 (94%) / 18 (6%)
Airway SGA / ETT	193 (95%) / 10 (5%)	300 (98%) / 6 (2%)
Airway removal timing		
Awake	152 (75%)	246 (80%)
Deep	51 (25%)	60 (20%)
Anesthetic Duration minutes, median (P25, P75)	42.0 (36, 51)	39.50 (34, 47)
Intraoperative opioid use	203 (100%)	306 (100%)
Intraoperative OME (mg.kg^-1^)	0.41 (0.13)	0.40 (0.12)
Fentanyl	129 (64%)	137 (45%)
Morphine	106 (52%)	75 (25%)
Hydromorphone	26 (14%)	25 (9%)
Pethidine	36 (19%)	138 (48%)
Tramadol	0 (0%)	4 (1%)
Remifentanil	0 (0%)	1 (0%)
Alfentanil	0 (0%)	2 (1%)
Premedication Midazolam	21 (10%)	32 (10%)
Premedication Clonidine	0	44 (14%)
Intraoperative Clonidine	0	264 (86%)
Dose in mcg.kg^-1^ (of those who had it)		0.98 (0.87, 1.12)
Unknown dose		22

Note: Clonidine information was missing for n = 3 participants – more information can be found in the Supplementary Material. Two patients received both premedication and intraoperative clonidine. SD, Standard Deviation; Kg, Kilogram; P25, 25^th^ percentile: P75, 75^th^ percentile; Cm, Centimeters; BMI, Body Mass Index; ASA, American Society of Anesthesiologists physical status classification; SGA, Supraglottic Airway; ETT, Endotracheal Tube; TIVA, Total Intravenous Anesthesia.

**Table 2:** Risk factors for perioperative respiratory adverse events according to clonidine status.

Characteristic	No Clonidine (n = 203)	Clonidine (n = 306)
URTI in 2 weeks preoperatively	31 (15%)	59 (19%)
Nocturnal dry cough	34 (17%)	59 (19%)
Wheeze during Exercise	38 (19%)	33 (11%)
Asthma	36 (18%)	43 (14%)
Hay fever	59 (29%)	87 (28%)
Eczema	52 (26%)	77 (25%)
Asthma – Family History	74 (36%)	94 (31%)
Eczema – Family History	61 (30%)	95 (31%)
Hay fever – Family History	97 (48%)	132 (43%)
Caregiver Smoking	60 (30%)	75 (25%)

Note: Clonidine information was missing for n = 3 participants – more information can be found in the Supplementary Material. URTI, Upper Respiratory Tract Infection.

In the raw dataset, a total of 94 patients (18%) experienced postoperative respiratory events (during emergence or in the PACU, see Table 3). Severe postoperative respiratory events (bronchospasm and laryngospasm) were uncommon, 9 (2%) patients and similar between groups. None of the 3 patients with missing clonidine data had any type of adverse respiratory event. In this raw data, 25% (50/203) of participants in the no-clonidine group experienced any postoperative respiratory events compared with 14% (44/306) of those who received any clonidine. In patients with only intraoperative clonidine, it was 33/264 (13%).

**Table 3:** Postoperative respiratory events incidence according to clonidine status for the entire cohort without adjustment.

Postoperative Respiratory Event Type	No Clonidine (n = 203)	Clonidine (n = 306)
Bronchospasm	0 (0%)	1 (0%)
Laryngospasm	5 (2%)	3 (1%)
Coughing	23 (11%)	18 (6%)
Desaturation less than 95%	20 (10%)	20 (7%)
Airway Obstruction	20 (10%)	22 (7%)
**Respiratory** E**vent Timing**		
Emergence	16 (8%)	11 (4%)
PACU	43 (21%)	38 (12%)
**Postoperative Severe Respiratory Event**	**5 (2%)**	**4 (1%)**
**Any Postoperative Respiratory Event**	**50 (25%)**	**44 (14%)**

Note: Participants may have had more than one type of respiratory event or at more than one timing. PACU, Post-Anesthesia Care Unit. Severe respiratory events include bronchospasm and laryngospasm.

Propensity score matching, narrowed the difference in postoperative respiratory event incidence between groups. In the complete case dataset, the incidence was 22% in the no-clonidine group and 17% in the clonidine group (OR = 0.732, 95% CI 0.420‒1.265), a reduction of 23%, and similarly in the imputed (m = 100) dataset (n = 347.4) of 4 percentage points (22% to 18%). No statistically significant effect of clonidine on the rate of postoperative respiratory events in the matched datasets was observed overall or at emergence or in PACU separately, with Odds Ratios (95% CI) ranging from 0.616 (0.361, 1.047) to 0.757 (0.429, 1.335) across models (Table 4). When included in the propensity score matching model intraoperative OME did not impact the results; this, coupled with the rate of missingness in intraoperative opioid dosing relative to other variables (Supplement Fig. 3) and the similar mean OME between clonidine and no-clonidine groups (Table 1), led to the decision not to include this as a predictor in the final model.

**Table 4:** Results from models (logistic regression for the outcome of Perioperative Respiratory Adverse Events (PRAE), linear regression on the log of Post-Anesthesia Care Unit (PACU) length of stay for the outcome of PACU length of stay) for raw complete-case, matched complete-case, and matched multiply imputed datasets.

RAW CCA	No Clonidine, n = 203	Clonidine, n = 306	Model estimate (95% CI)	p-value
PRAE at emergence and/or PACU	50 (25%)	44 (14%)	0.616 (0.361, 1.047)^a^	0.074^a^
PRAE at Emergence	16 (7.9%)	11 (3.6%)	0.466 (0.180, 1.162)^a^	0.104^a^
PRAE at PACU	43 (21%)	38 (12%)	0.675 (0.386, 1.176)^a^	0.166^a^
Time in PACU (mins), median (P25‒P75)	44.0 (33.0‒58.8)	45.0 (35.0‒59.0)	0.023 (-0.05, 0.097)^c^	0.533^a^
**Matched CCA**	**No Clonidine, n = 163**	**Clonidine, n = 163**		
PRAE at emergence and/or PACU	36 (22%)	28 (17%)	0.732 (0.420, 1.265)^b^	0.266^b^
PRAE at Emergence	11 (6.7%)	9 (5.5%)	0.808 (0.317, 2.006)^b^	0.645^b^
PRAE at PACU	30 (18%)	23 (14%)	0.728 (0.399, 1.314)^b^	0.295^b^
Time in PACU mins, median (P25‒P75)	45.0 (34.0‒59.0)	45.0 (35.0‒57.0)	0.036 (-0.054, 0.126)^c^	0.434^c^
**Matched Imputed**	**No Clonidine, n = 173.7**	**Clonidine, n = 173.7**		
PRAE at emergence and/or PACU	38.8 (22%)	31.2 (18%)	0.757 (0.429, 1.335)^b^	0.334^b^
PRAE at Emergence	10.5 (6.1%)	8.2 (4.7%)	0.754 (0.275, 2.067)^b^	0.582^b^
PRAE at PACU	34.0 (20%)	27.1 (16%)	0.757 (0.419, 1.368)^b^	0.354^b^
Time in PACU (mins), median (P25‒P75)	44.3 (33.9‒59.0)	44.7 (34.8‒59.1)	0.025 (-0.070, 0.121)^c^	0.601^c^

Notes:

a Raw OR and p-values adjusted for significant confounders, including patient characteristics and surgical information via a multivariable model.

b Matched OR and p-values for single regression models since confounders are handled in propensity score matching process prior to modelling.

c Model estimates and p-values are from linear regression models for log-transformed length of stay, while the medians are reported on the original linear scale. OR, Odds Ratio; CI, Confidence Interval; Est, Parameter Estimate; CCA, Complete Case Analysis.

For the matched complete case data, the mean length of stay in PACU was 48.2 minutes (SD ±22.5 minutes), compared with 50.0 minutes (SD ±23.5 minutes) for those receiving clonidine. Based on inspection of residual distributions, the PACU duration was log-transformed before linear modelling. Based on these models, there was no significant difference in the mean duration of stay in PACU between groups in any analysis (Table 4).

## Discussion

In this post-hoc propensity-matched analysis, pre- or intraoperative clonidine was not associated with a statistically significant reduction in the incidence of postoperative respiratory events in children undergoing adenotonsillectomy. Although a lower incidence of respiratory events was observed in the clonidine group in the raw data (14% vs. 25%), when correcting for baseline differences and accounting for missing data this difference narrowed (18% vs. 22%; OR = 0.757 [95% CI 0.43‒1.34]) and remained not significant (Table 4). The overall incidence of postoperative respiratory events reported in our cohort was 18%, which is consistent and at the lower end of studies in the literature.^23^, ^24^, ^25^ This low rate may in part be due to the use of flexible SGAs rather than endotracheal tubes as standard airway management in our institution.^26^ In addition, clonidine use did not seem to prolong the time to PACU discharge.

An animal model has shown the potential beneficial effects of alpha-2 agonists on the respiratory system, which may be due to an increased depth of anesthesia, blunting airway reflexes, a reduction in airway reactivity, increased bronchodilation and cough suppression.^27^ Importantly, oral clonidine premedication at 4 mcg.kg^-1^ does not seem to attenuate the carbon dioxide response curve in children undergoing sevoflurane anesthesia,^28^ which, with its analgesic, anxiolytic and sedative actions, allows sparing or reduction in midazolam, sevoflurane and opioids, perhaps promoting a better wake-up from anesthesia.

Alpha-2 agonists have demonstrated a benefit in those at high risk of adverse respiratory events, for example in those with OSA, reactive or inflamed airways. Shen et al.^8^ reported significantly lower respiratory adverse events rates in children, prior to tonsillectomy, receiving premedication with intranasal dexmedetomidine (24%) compared to midazolam (56%) or saline (40%). In their post-hoc analysis, there was an even more pronounced effect in those with URTI in the preceding 4 weeks, 20.0% had respiratory events with dexmedetomidine vs. 64.4% in the midazolam group. Another RCT of children with congenital heart disease and recent upper respiratory tract infection, undergoing cardiac catheterization, showed that intranasal dexmedetomidine more than halved the incidence of adverse respiratory events in those aged less than 3 years.^7^ A meta-analysis of dexmedetomidine studies from ten older pediatric studies containing 1,056 patients, with four of the studies in tonsillectomy patients, demonstrated reduced PRAE compared to active comparators and reduced cough, breathing holding and laryngospasm compared to placebo.^29^ Preoperative midazolam may be associated with increased PRAE,^12^ but was spread evenly across groups.

Our data has several limitations. The retrospective nature of our analysis and merged data from past trials over a four-year period introduce potential biases, particularly reliance on data that was not originally collected for this research question, as well as changes in anesthesia or surgical technique over time. The raw data for the clonidine group (Table 2) had several factors, e.g. more private patients, more patients from the more recent BEE study, more males, less obese patients, higher use of TIVA and SGAs, many of which may be protective for respiratory events^14^^,^^30^ but were adjusted for in the analysis, and may explain the narrowing in incidence in the matched data set and did not demonstrate benefit. It may be that the sample size available for the adjusted analyses was insufficient to exclude modest effect sizes with precision.

The MICE analysis allowed for missing data and showed a similar result, adding to the statistical reliability of the findings. Despite the propensity score matching and MICE, residual confounding might still play a role, particularly due to patient factors that were not recorded or standardized, for example doses and types of opioids used across studies and documentation of local anesthetic or exact hemostatic technique used by surgeons was not recorded in studies.

This confounding may also include the anesthesiologist’s reasons for using clonidine, for example, an anxious child may have a more stormy IV induction without preoxygenation, leading to increased risk of desaturation. Of note, for the 14% receiving preoperative clonidine the dose was not recorded in the original studies, so only a binary analysis of “clonidine or no clonidine” was used; additionally, propensity score matched analysis is only valid for a binary exposure. We did not explore whether variations in clonidine dosing impacted rates of respiratory events differently, due to the post-hoc nature of the analysis. It is possible that different doses or timing of doses could have different effects. That said, looking just at intraoperative intravenous dosing (86% of cases), which was largely consistent at 1 μg.kg^-1^, the rate of respiratory events was similar in the raw data to those receiving preoperative dosing, despite the higher doses given. Another limitation is the lack of standardization of anesthesia, including induction technique, drug selection (opioid) and dosing, as well as extubation strategies. All of these differences can affect the incidence of adverse respiratory events,^14^^,^^30^ but they were also accounted for in the propensity score matched modelling and represent a more “real-world” anesthesia approach. Predominantly, the anesthesia technique was an inhalational induction and maintenance with sevoflurane. Propofol-based TIVA was used slightly more in the clonidine group (6% vs. 1%), which has been associated with fewer adverse respiratory events.^25^^,^^31^ However, even prior to matching the numbers of propofol, TIVA instances were statistically balanced between clonidine and no-clonidine groups (Supplement Figs. 6 and 8). Of note, in our institutions, a flexible supraglottic airway is commonly used during tonsillectomy, 96.8% of this cohort. The use of supraglottic airway has been shown to reduce the incidence of adverse respiratory events^12^^,^^14^^,^^30^ compared to endotracheal intubation, and was used slightly more in the non-clonidine group. In particular, SGA versus ETT for tonsillectomy in children has shown to nearly halve respiratory adverse events, with Odds Ratio (OR = 0.55 [95% CI: 0.36, 0.82], p = 0.004) but with a similar incidence of laryngospasm (OR = 0.84 [95% CI: 0.39, 1.81]).^26^ It is likely that the high use of SGA may have lowered the base incidence rate in both groups. Many institutions use endotracheal tubes as standard for tonsillectomy, which limits generalizability beyond SGA case tonsillectomy. Despite minor variations in anesthesia conduct, the study had patients across four centers, both public and private, with different surgeons and anesthesiologists which adds to the generalizability of the study.

It should also be noted that recent URTI, one of the most important risk factors for adverse respiratory events, was more prevalent in the clonidine group (19% vs. 15%, see Table 2). This was also adjusted for via propensity score matching, resulting in rates of 14% URTI in the matched clonidine group versus 15% in the no-clonidine group. The vast majority of cases were performed to treat sleep-disordered breathing/snoring – a known risk factor for adverse respiratory events. In particular, the severity of any associated Obstructive Sleep Apnea (OSA) was not known between the groups (apart from the OSATS2 patients), as polysomnography is not routinely performed preoperatively even though OSA severity represents an important confounding risk factor.^32^

While this study focused on clonidine, it is worth considering that dexmedetomidine’s greater selectivity for alpha-2-receptors, used at similar dosing (1‒2 μg.kg^-1^), could account for a superior efficacy in the prior animal and clinical studies.^7^^,^^8^ A relatively low dose of clonidine (1 μg.kg^-1^) was used in most patients, and it may be that a higher dose, such that it approximates an equipotent dose of dexmedetomidine, might be required. While we did not find a significant reduction in postoperative respiratory events, clonidine use did not increase PACU time at this dose. This is of particular importance since clonidine is widely used in clinical practice and is inexpensive. There is a gap for a high-quality prospective randomized controlled trial, preferably using differing doses of clonidine perioperatively, to definitely investigate its role in reducing respiratory adverse events. This is particularly true for research in high-risk subgroups, for example, in those with a recent URTI or proven OSA or at institutions that use predominantly ETTs, since those children might gain more from clonidine.

## Conclusion

This post-hoc analysis found no evidence of a difference in postoperative respiratory events when administering perioperative clonidine in pediatric patients undergoing extracapsular (adeno)tonsillectomy with a flexible supraglottic airway. Clonidine at doses used in this cohort did not prolong time in PACU.

## Trial registration

This post-hoc analysis includes data from three prospective research projects on children undergoing elective (adeno-) tonsillectomies between January 2018 and November 2021: the Bee Pain Free (Child and Adolescent Health Service Human Research Ethics Committee Approval CAHS HREC RGS0000003325, Australia and New Zealand Clinical Trials Registry ACTRN12619001385134), EMPHASIS (CAHS RGS0000000296; ACTRN12617001379303), and OSATS2 (CAHS RGS0000000014; ACTRN12615001037594)

## AI-use disclosure

AI has not been used in the preparation of this manuscript.

## Data availability statement

The data for this manuscript are available upon reasonable request and in accordance with institutional ethical and governance requirements.

## CRediT authorship contribution statement

**David Sommerfield:** Conceptualization, Funding acquisition, Investigation, Methodology, Writing – original draft, Writing – review & editing. **Bojana Stepanovic:** Investigation, Writing – original draft, Writing – review & editing. **Daisy Evans:** Conceptualization, Data curation, Formal analysis, Methodology, Writing – review & editing. **Andy Jeon:** Investigation, Writing – review & editing. **Anne Louise de Barros Garioud:** Investigation, Writing – review & editing. **Aine Sommerfield:** Conceptualization, Funding acquisition, Project administration, Writing – review & editing. **Neil Hauser:** Conceptualization, Funding acquisition, Methodology, Writing – review & editing. **R. Nazim Khan:** Conceptualization, Formal analysis, Supervision, Writing – review & editing. **Britta S. von Ungern-Sternberg:** Conceptualization, Funding acquisition, Methodology, Investigation, Project administration, Resources, Supervision, Writing – review & editing.

## Conflicts of interest

The authors declare no conflicts of interest.

## Appendix Supplementary materials

Image, application 1

## Footnotes

Supplementary material associated with this article can be found, in the online version, at doi:10.1016/j.bjane.2026.844792.
