# Supplementary material for: Impact of perioperative clonidine on postoperative respiratory adverse events in children: a post-hoc observational analysis of prospectively collected datasets
Source: Braz J Anesthesiol. 2026 Jul 8;76(5):844792. doi: 10.1016/j.bjane.2026.844792 (PMC13449382; doi:10.1016/j.bjane.2026.844792)
Supplement: Supplementary file 1 [file mmc1.docx]

**BJAN-D-25-00890_ Supplementary Material**

**Clonidine & Postoperative Respiratory Events Supplementary Material**

Contents

[1	Study Background	1](#_Toc203307156)

[2	Statistical Methods	1](#_Toc203307157)

[3	Data Analysis	2](#_Toc203307158)

[3.1	Characterising missing data	6](#_Toc203307159)

[3.2	Multiple Imputations by Chained Equations (MICE)	9](#_Toc203307160)

[3.3	Estimating Propensity Scores & Matching	11](#_Toc203307161)

[3.3.1	Matching with complete cases	12](#_Toc203307162)

[3.3.2	Matching with m=100 imputations	16](#_Toc203307163)

[3.4	Analysis of matched datasets	17](#_Toc203307164)

[3.4.1	PRAE at Emergence and/or PACU	17](#_Toc203307165)

[3.4.2	PRAE at Emergence	18](#_Toc203307166)

[3.4.3	PRAE at PACU	18](#_Toc203307167)

[3.4.4	PACU Length of stay	19](#_Toc203307168)

[3.5	Multivariable model on Raw Cohort	21](#_Toc203307169)

[4	References	22](#_Toc203307170)

# 1 Study Background

The primary aim is to assess whether giving children clonidine pre- or intraoperatively reduces the occurrence of perioperative respiratory adverse events (PRAE) during the emergence and recovery period. Secondary outcomes are the occurrence of PRAE during emergence alone, recovery alone, and the length of time from arrival in the post-anesthesia care unit (PACU) to the time when the patient is ready for discharge.

Given that this is a retrospective observational study, propensity score matching (PSM) will be used to account for any systematic differences between patients who were and were not given clonidine pre- or intraoperatively.

# 2 Statistical Methods

All analyses will be performed in the R statistical environment.^1^ Statistical significance will be taken at 5% (p<0.05) unless otherwise stated.

Baseline variables for the whole cohort before matching will be summarized between clonidine and no-clonidine groups using summary statistics and graphical methods. The proportion of missing values in each variable of interest will also be considered. In the case that missingness rates are high, multiple imputation by chained equations (MICE)^2,3^ will be applied, and outcomes for the Complete Case Analysis (CCA) and multiply imputed analyses will be compared.

Propensity scores (PS) will be estimated using a logistic regression model, with the dependent variable being treatment with clonidine pre- and/or intraoperatively. True confounders (variables related to both treatment with clonidine and outcomes of interest) and variables related to the outcomes but not the treatment should be included in the PS estimation model.^4^ Unless it is known that a variable is not related to the outcome, it will be included in the model for PS regardless of significance level.^5^ Mediating variables (in the causal path from treatment to outcome) and variables related only to the treatment but not to the outcome will not be included as covariates in the PS model.^6,4^ The overlap in distributions of PS between treatment and control groups will be assessed by considering density plots.

The logit of propensity scores will be matched between clonidine and no-clonidine cohorts using a 1:1 nearest neighbor approach without replacement, and with a caliper of size 0.2*standard deviation of the logit of the estimated PS. The proportion of successfully matched participants in each group will be considered to assess the suitability of the matching algorithm. Balance diagnostics will be assessed, including standardized mean differences (SMD), graphical summaries, and variance ratios (VR). If MICE has been used, PS models will be fit to each completed dataset, followed by fitting the analysis model, and the results of analysis models will be pooled.^6,7^

To estimate the effect of treatment with clonidine on the incidence of PRAE, logistic regression models will be fitted to the binary (yes or no) outcomes of PRAE at emergence, PACU, and emergence and/or PACU combined, for both the CCA and MI datasets. Baseline covariates which showed residual differences between treatment groups following PSM will be included in the model for PRAE. Results for the CCA models and MI models will be considered and compared.

To estimate the effect of treatment with clonidine on the length of stay in PACU, a linear model will be fit to the continuous outcome of PACU duration (in minutes) for CCA and MI datasets. Variance structures will be considered based on model diagnostics.

# 3 Data Analysis

Figure 1 shows the date and age at surgery of patients included in this cohort, colored by the study in which they were enrolled. The youngest participant was 1 year and 4 months, and the oldest was 16 years and 5 months. There are clear gaps in recruitment over COVID-19 lockdown periods in Perth.

**Figure 1** Recruitment of participants over time with their age and which study they were enrolled in.

Of the 512 patients in these three studies who had tonsillectomy, 3 have missing data on whether they had clonidine pre- and/or intraoperatively. Although there were small numbers in some sub-groups (e.g. patients having ETT airway device), exclusions were not made due to the complexity of applying exclusion/inclusion criteria with MICE where the criterion variable has missing data.^8^ Table 1 shows some characteristics of patients with respect to clonidine administered pre- or intraoperatively. Table 2 summarizes the PRAE incidence in the total cohort. Figure 2 shows the distribution of PACU duration between clonidine groups. At first glance, there is no obvious difference in PACU lengths of stay between the two groups.

**Table 1** Baseline and surgical characteristics of patients grouped by whether or not they received clonidine pre- and/or intraoperatively. Percentages are given column-wise.

| **Characteristic** | **No (Pre or Intra) Clonidine (**N = 203) | **Yes (Pre or Intra) Clonidine (**N = 306) | **Missing (pre or Intra) clonidine (N = 3)** | **Overall (N = 512)** |
| --- | --- | --- | --- | --- |
| Trial |  |  |  |  |
| BEE | 141 (69%) | 249 (81%) | 0 (0%) | 390 (76%) |
| EMPHASIS | 22 (11%) | 35 (11%) | 3 (100%) | 60 (12%) |
| OSATS2 | 40 (20%) | 22 (7.2%) | 0 (0%) | 62 (12%) |
| YearOfSurgery |  |  |  |  |
| 2018 | 14 (6.9%) | 18 (5.9%) | 1 (33%) | 33 (6.4%) |
| 2019 | 36 (18%) | 22 (7.2%) | 0 (0%) | 58 (11%) |
| 2020 | 123 (61%) | 188 (61%) | 2 (67%) | 313 (61%) |
| 2021 | 30 (15%) | 78 (25%) | 0 (0%) | 108 (21%) |
| Site |  |  |  |  |
| Public | 185 (91%) | 198 (65%) | 0 (0%) | 383 (75%) |
| Private | 18 (8.9%) | 108 (35%) | 3 (100%) | 129 (25%) |
| Age, mean (sd) | 6.70 (2.97) | 6.23 (2.95) | 7.26 (2.71) | 6.42 (2.96) |
| Age.f |  |  |  |  |
| 0 to 5.9 | 95 (47%) | 162 (53%) | 1 (33%) | 258 (50%) |
| 6 to 8.9 | 75 (37%) | 99 (32%) | 1 (33%) | 175 (34%) |
| 9 and over | 33 (16%) | 45 (15%) | 1 (33%) | 79 (15%) |
| WeightKG, median (LQ,UQ) | 22.95 (19.15, 31.80) | 21.26 (16.80, 28.25) | 27.00 (17.20, 40.00) | 21.80 (17.65, 29.45) |
| HeightCM, mean (sd) | 121.87 (17.72) | 117.69 (19.86) | 129.67 (20.84) | 119.47 (19.11) |
| Unknown | 7 | 24 | 0 | 31 |
| BMI, median (UQ, LQ) | 16.35 (15.27, 19.25) | 16.40 (15.30, 19.00) | 15.00 (15.00, 18.30) | 16.40 (15.30, 19.00) |
| Unknown | 7 | 24 | 0 | 31 |
| BMI.zscore, mean (sd) | 0.54 (1.35) | 0.45 (1.49) | -0.08 (0.82) | 0.48 (1.43) |
| Unknown | 8 | 32 | 0 | 40 |
| ASA |  |  |  |  |
| I | 81 (40%) | 135 (44%) | 2 (67%) | 218 (43%) |
| II | 120 (59%) | 167 (55%) | 1 (33%) | 288 (56%) |
| III | 2 (1.0%) | 4 (1.3%) | 0 (0%) | 6 (1.2%) |
| Sex |  |  |  |  |
| Male | 125 (62%) | 153 (50%) | 2 (67%) | 280 (55%) |
| Female | 78 (38%) | 153 (50%) | 1 (33%) | 232 (45%) |
| Induction |  |  |  |  |
| IV | 28 (14%) | 31 (10%) | 0 (0%) | 59 (12%) |
| Inhalation | 175 (86%) | 275 (90%) | 3 (100%) | 453 (88%) |
| Airway |  |  |  |  |
| LMA | 193 (95%) | 300 (98%) | 3 (100%) | 496 (97%) |
| ETT | 10 (4.9%) | 6 (2.0%) | 0 (0%) | 16 (3.1%) |
| Airway removal timing |  |  |  |  |
| Awake | 152 (75%) | 246 (80%) | 3 (100%) | 401 (78%) |
| Deep | 51 (25%) | 60 (20%) | 0 (0%) | 111 (22%) |
| Maintenance |  |  |  |  |
| Gas | 200 (99%) | 288 (94%) | 3 (100%) | 491 (96%) |
| TIVA | 3 (1.5%) | 18 (5.9%) | 0 (0%) | 21 (4.1%) |
| AnestheticDuration, median (LQ,UQ) | 42.00 mins (36.00 mins, 51.00 mins) | 39.50 mins (34.00 mins, 47.00 mins) | 30.00 mins (30.00 mins, 30.00 mins) | 40.00 mins (35.00 mins, 49.00 mins) |
| Unknown | 1 | 2 | 2 | 5 |
| PACUDuration, median (LQ, UQ) | 44.00 (33.00, 59.00) | 45.00 (35.00, 59.00) | 48.00 (48.00, 48.00) | 45.00 (35.00, 59.00) |
| Unknown | 2 | 3 | 2 | 7 |
| Wheeze | 41 (20%) | 52 (17%) | 0 (0%) | 93 (18%) |
| Cough | 34 (17%) | 59 (19%) | 1 (33%) | 94 (18%) |
| ColdOrFlu | 31 (15%) | 59 (19%) | 2 (67%) | 92 (18%) |
| Unknown | 0 | 1 | 0 | 1 |
| WheezeExercise | 38 (19%) | 33 (11%) | 0 (0%) | 71 (14%) |
| PreviousAsthma | 36 (18%) | 43 (14%) | 0 (0%) | 79 (15%) |
| Hayfever | 59 (29%) | 87 (28%) | 1 (33%) | 147 (29%) |
| Eczema | 52 (26%) | 77 (25%) | 1 (33%) | 130 (25%) |
| Unknown | 0 | 1 | 0 | 1 |
| AsthmaGT2Family | 74 (36%) | 94 (31%) | 0 (0%) | 168 (33%) |
| EczemaGT2Family | 61 (30%) | 95 (31%) | 1 (33%) | 157 (31%) |
| HayfeverGT2Family | 97 (48%) | 132 (43%) | 1 (33%) | 230 (45%) |
| CaregiverSmoking | 60 (30%) | 75 (25%) | 0 (0%) | 135 (26%) |

**Table 2** PRAE incidence for patients who did and did not receive pre- and/or intraoperative clonidine. Participants may have had more than one type of PRAE or at more than one timing.

| **PRAE** | **No Pre or Intra Clonidine (**N = 203) | **Yes Pre or Intra Clonidine (**N = 306) | **Missing pre or intra clonidine (N = 3)** | **Overall (N = 512)** |
| --- | --- | --- | --- | --- |
| **PRAE Type** |  |  |  |  |
| AnyBronchospasm | 0 (0%) | 1 (0.3%) | 0 (0%) | 1 (0.2%) |
| AnyLaryngospasm | 7 (3.4%) | 5 (1.6%) | 0 (0%) | 12 (2.3%) |
| AnyCoughing | 24 (12%) | 20 (6.5%) | 0 (0%) | 44 (8.6%) |
| AnyDesatsLT95 | 21 (10%) | 25 (8.2%) | 0 (0%) | 46 (9.0%) |
| AnyAirObs | 21 (10%) | 28 (9.2%) | 0 (0%) | 49 (9.6%) |
| **PRAE Timing** |  |  |  |  |
| AnyInduction | 7 (3.4%) | 20 (6.5%) | 0 (0%) | 27 (5.3%) |
| AnyMaintenance | 5 (2.5%) | 2 (0.7%) | 0 (0%) | 7 (1.4%) |
| AnyEmergence | 16 (7.9%) | 11 (3.6%) | 0 (0%) | 27 (5.3%) |
| AnyPACU | 43 (21%) | 38 (12%) | 0 (0%) | 81 (16%) |
| **Postoperative PRAE Types** |  |  |  |  |
| Bronchospasm | 0 (0%) | 1 (0%) | 0 (0%) | 1 (0%) |
| Laryngospasm | 5 (2%) | 3 (1%) | 0 (0%) | 8 (2%)) |
| Coughing | 23 (11%) | 18 (6%) | 0 (0%) | 41 (8%) |
| Desaturation less than 95% | 20 (10%) | 20 (7%) | 0 (0%) | 40 (8%) |
| Airway Obstruction | 20 (10%) | 22 (7%) | 0 (0%) | 44 (9%) |
| **AnySeverePRAE** | 7 (3.4%) | 6 (2.0%) | 0 (0%) | 13 (2.5%) |
| **AnyEmergencePACUPRAE** | 50 (25%) | 44 (14%) | 0 (0%) | 94 (18%) |
| **AnyPRAE** | 53 (26%) | 53 (17%) | 0 (0%) | 106 (21%) |

**Figure 2** Distribution of PACU lengths of stay in groups of patients who did or did not have clonidine.

## 3.1 Characterizing missing data

Figure 3 shows the proportion of missing data for variables of interest. The missingness in post-op clonidine is very high – too high to impute using MICE – so we omit it from the dataset. We will also not include BMI z-score or percentile because they do not exist for children under 2 years old. We will also not consider actual doses of pre- or intraoperative clonidine or midazolam, because when considering only those who were marked as having had certain drugs, the missingness rate is actually very high (most who had drugs did not have a dose recorded). Intraoperative opioids, and the intraoperative OME, will also not be considered in models. Figure 4 shows the missing data pattern with these variables removed.

**Figure 3** Percent of missing values in each variable. Variables with no missing values are not shown.

**Figure 4** Missing data pattern for the variables of interest in the dataset. Pink boxes indicate missing data. The variable names are shown above the columns, with the numbers of missing values in each of these variables shown below. The number of records with each pattern of missing data are shown to the left of the rows. The number of missing values in each pattern are shown to the right of the rows. Only variables with at least one missing value are shown in this figure for brevity.

471 (92%) of the participants have no missing values in any of the variables of interest. From Table 3, it’s clear that there are differences between the cohorts of patients who have complete datasets and those who don’t.

**Table 3** Patient characteristics for those with no missing values compared with those with any missing values. Percentages are given column-wise.

| **Characteristic** | **No Missing Values**, N = 471 | **Any Missing Values**, N = 41 |
| --- | --- | --- |
| AnyClonidine.PreOrIntra | 277 (59%) | 29 (76%) |
| Missing | 0 | 3 |
| Trial |  |  |
| BEE | 361 (77%) | 29 (71%) |
| EMPHASIS | 51 (11%) | 9 (22%) |
| OSATS2 | 59 (13%) | 3 (7%) |
| YearOfSurgery |  |  |
| 2018 | 29 (6%) | 4 (10%) |
| 2019 | 57 (12%) | 1 (2%) |
| 2020 | 281 (60%) | 32 (78%) |
| 2021 | 104 (22%) | 4 (10%) |
| Site |  |  |
| Public | 370 (79%) | 13 (32%) |
| Private | 101 (21%) | 28 (68%) |
| Age | 6.51 (3.00) | 5.35 (2.29) |
| Age.f |  |  |
| 0 to 5.9 | 233 (49%) | 25 (61%) |
| 6 to 8.9 | 161 (34%) | 14 (34%) |
| 9 and over | 77 (16%) | 2 (5%) |
| WeightKG | 22.00 (17.94, 30.20) | 19.95 (16.50, 25.50) |
| HeightCM | 119.35 (19.16) | 124.97 (16.19) |
| Missing | 0 | 31 |
| BMI | 16.40 (15.30, 19.10) | 15.90 (15.00, 18.30) |
| Missing | 0 | 31 |
| ASA |  |  |
| 1 | 203 (43%) | 15 (37%) |
| 2 | 263 (56%) | 25 (61%) |
| 3 | 5 (1%) | 1 (2%) |
| Sex |  |  |
| Male | 252 (54%) | 28 (68%) |
| Female | 219 (46%) | 13 (32%) |
| PreMedMidaz.y | 52 (11%) | 2 (5%) |
| Missing | 0 | 2 |
| IntraopOME_perkg | 0.40 (0.12) | 0.42 (0.11) |
| Missing | 0 | 31 |
| Induction |  |  |
| IV | 57 (12%) | 2 (5%) |
| Inhalation | 414 (88%) | 39 (95%) |
| Airway |  |  |
| LMA | 456 (97%) | 40 (98%) |
| ETT | 15 (3%) | 1 (2%) |
| Maintenance |  |  |
| Gas | 454 (96%) | 37 (90%) |
| TIVA | 17 (4%) | 4 (10%) |
| Airway removal |  |  |
| Awake | 369 (78%) | 32 (78%) |
| Deep | 102 (22%) | 9 (22%) |
| AnestheticDuration | 40.00 mins (35.00 mins, 49.00 mins) | 38.00 mins (30.00 mins, 43.00 mins) |
| Missing | 0 | 5 |
| PACUDuration | 45.00 (35.00, 59.00) | 44.00 (30.00, 59.00) |
| Missing | 0 | 7 |
| Wheeze | 84 (18%) | 9 (22%) |
| Cough | 87 (18%) | 7 (17%) |
| ColdOrFlu | 81 (17%) | 11 (28%) |
| Missing | 0 | 1 |
| WheezeExercise | 68 (14%) | 3 (7%) |
| PreviousAsthma | 74 (16%) | 5 (12%) |
| Hayfever | 137 (29%) | 10 (24%) |
| Eczema | 120 (25%) | 10 (25%) |
| Missing | 0 | 1 |
| AsthmaGT2Family | 154 (33%) | 14 (34%) |
| EczemaGT2Family | 141 (30%) | 16 (39%) |
| HayfeverGT2Family | 215 (46%) | 15 (37%) |
| CaregiverSmoking | 127 (27%) | 8 (20%) |
| TotalRF | 2.00 (1.00, 4.00) | 2.00 (1.00, 4.00) |
| Missing | 0 | 1 |
| AnyPRAE | 96 (20%) | 10 (24%) |
| AnyEmergPRAE | 25 (5%) | 2 (5%) |
| AnyPACUPRAE | 71 (15%) | 10 (24%) |
| AnyEmergPACUPRAE | 84 (18%) | 10 (24%) |
| AnySeverePRAE | 13 (3%) | 0 (0%) |

## 3.2 Multiple Imputations by Chained Equations (MICE)

To determine which predictors should be included in the MICE model for each variable with missingness, we need to consider correlations with the value of the variable, and correlations with the variable’s missingness. For each variable with any missingness, Table 4 shows the variables which are correlated with magnitude > 0.3 with its value and its likelihood of being missing. Some have no correlating variables.

**Table 4** Summary of which variables correlate with the value and missingness flag for each variable of interest.

| **Variable** | **N missing** | **Correlation with value > 0.3** | **Correlation with missing > 0.3** |
| --- | --- | --- | --- |
| PreMedMidaz.y | 2 |  |  |
| AnyClonidine.PreOrIntra | 3 |  |  |
| ColdOrFlu | 1 |  |  |
| Eczema | 1 | TotalRF |  |
| TotalRF | 1 | AsthmaGT2Family, EczemaGT2Family, WheezeExercise, HayfeverGT2Family, Wheeze, PreviousAsthma, Hayfever, Eczema, CaregiverSmoking, Cough |  |
| HeightCM | 31 | Age, WeightKG, Induction, BMI |  |
| BMI | 31 | WeightKG, Age, HeightCM, Induction |  |
| AnestheticDuration | 5 |  |  |
| PACUDuration | 7 |  |  |

Start by considering which variables are relevant and need to be imputed or included in the MICE models as predictors. All the risk factors have all the other risk factors as predictors. Predictors based on correlation magnitude > 0.3, and predictors of predictors, are used to begin with. If no predictors were found for a given variable, appropriate theoretical predictors were used. We will also use the outcome (AnyEmergPACUPRAE, AnySeverePRAE, AnyEmergence, AnyPACU, PACUDuration) as predictors for all these variables (unless they are being predicted by passive imputation). The maximum number of predictors for any one imputation model is 16.

**Table 5** Missingness and MICE model information for all variables considered.

| **Variable** | **N Missing** | **Imputation Method** | **Predictors** |
| --- | --- | --- | --- |
| PreMedMidaz.y | 2 | pmm | WeightKG, Age, HeightCM, AnyClonidine.PreOrIntra, AnyPRAE, AnySeverePRAE, AnyEmergPACUPRAE, AnyEmergence, AnyPACU, PACUDuration |
| AnyClonidine.PreOrIntra | 3 | pmm | WeightKG, Age, HeightCM, PreMedMidaz.y,, Trial, Site.cat, AnyPRAE, AnySeverePRAE, AnyEmergPACUPRAE, AnyEmergence, AnyPACU, PACUDuration |
| ColdOrFlu | 1 | pmm | Wheeze, Cough, WheezeExercise, PreviousAsthma, Hayfever, Eczema, AsthmaGT2Family, EczemaGT2Family, HayfeverGT2Family, CaregiverSmoking, AnyPRAE, AnySeverePRAE, AnyEmergPACUPRAE, AnyEMergence, AnyPACU, PACUDuration |
| TotalRF | 8 | ~I((Wheeze=="YES")+ (Cough=="YES")+ (ColdOrFlu=="YES")+ (WheezeExercise=="YES")+ (PreviousAsthma=="YES")+ (Hayfever=="YES")+ (Eczema=="YES")+ (AsthmaGT2Family=="YES")+  (EczemaGT2Family=="YES")+ (HayfeverGT2Family=="YES")+ (CaregiverSmoking=="YES")) | Wheeze, Cough, ColdOrFlu, WheezeExercise, PreviousAsthma, Hayfever, Eczema, AsthmaGT2Family, EczemaGT2Family, HayfeverGT2Family, CaregiverSmoking |
| HeightCM | 31 | pmm | Age, WeightKG, Induction, Sex, AnyPRAE, AnySeverePRAE, AnyEmergPACUPRAE, AnyEMergence, AnyPACU, PACUDuration |
| BMI | 32 | ~I(WeightKG/HeightCM^2) | WeightKG, HeightCM |
| AnaestheticDuration | 5 | pmm | WeightKG, HeightCM, Age, YearOfSurgery, Site, AnyPRAE, AnySeverePRAE, AnyEmergPACUPRAE, AnyEMergence, AnyPACU, PACUDuration |
| PACUDuration | 7 | pmm | Trial, YearOfSurgery, WeightKG, HeightCM, Site, AnyPRAE, AnySeverePRAE, AnyEmergPACUPRAE, AnyEMergence, AnyPACU, PACUDuration |

Details on the imputation diagnostics are omitted here for brevity.

## 3.3 Estimating Propensity Scores & Matching

It is expected that the probability of experiencing PRAE is related to **respiratory risk factors** (history of respiratory symptoms, recent upper respiratory tract infection, eczema, family history of asthma and/or eczema, exposure to tobacco smoke, history of wheezing), **age** (risk decreases for older children), **ASA** category, **type of anesthesia induction** (inhalational induction increases risk relative to IV), **maintenance**, **airway device** (risk increased for ETT relative to LMA), and timing of **removal of airway device** (risk increased for removal while deep relative to awake).^9^ None of these variables are likely to be mediating variables in the causal path from clonidine to the outcome of PRAE, therefore all will be used in the propensity score estimating model.

Table 6 shows the distribution of patient characteristics between patients who did and did not experience PRAE at emergence and/or PACU.

All of the variables which have an expected/theoretical relationship with the outcome of PRAE will be included in the PS model, regardless of any actual relationship observed in the cohort.

**Table 6** Patient and surgical characteristics between groups of patients who did or did not have PRAE at emergence and/or PACU. Complete-case data (not imputed) is shown. Percentages are shown column-wise.

| **Characteristic** | **No PRAE**, N = 418^1^ | **PRAE**, N = 94^1^ |
| --- | --- | --- |
| AnyClonidine.PreOrIntra | 262 (63%) | 44 (47%) |
| Missing | 3 | 0 |
| Trial |  |  |
| BEE | 334 (80%) | 56 (60%) |
| EMPHASIS | 51 (12%) | 9 (9.6%) |
| OSATS2 | 33 (7.9%) | 29 (31%) |
| YearOfSurgery |  |  |
| 2018 | 24 (5.7%) | 9 (9.6%) |
| 2019 | 35 (8.4%) | 23 (24%) |
| 2020 | 260 (62%) | 53 (56%) |
| 2021 | 99 (24%) | 9 (9.6%) |
| Site |  |  |
| Public | 299 (72%) | 84 (89%) |
| Private | 119 (28%) | 10 (11%) |
| Age | 6.65 (3.02) | 5.40 (2.48) |
| Age.f |  |  |
| 0 to 5.9 | 196 (47%) | 62 (66%) |
| 6 to 8.9 | 148 (35%) | 27 (29%) |
| 9 and over | 74 (18%) | 5 (5.3%) |
| WeightKG | 22.30 (18.20, 30.94) | 19.80 (16.50, 26.40) |
| HeightCM | 120.77 (19.57) | 113.58 (15.68) |
| Unknown | 24 | 7 |
| BMI | 16.40 (15.20, 19.30) | 16.62 (15.40, 18.30) |
| Unknown | 25 | 7 |
| BMI.zscore | 0.47 (1.44) | 0.55 (1.39) |
| Unknown | 32 | 8 |
| ASA |  |  |
| I | 184 (44%) | 34 (36%) |
| II | 230 (55%) | 58 (62%) |
| III | 4 (1.0%) | 2 (2.1%) |
| Sex |  |  |
| Male | 221 (53%) | 59 (63%) |
| Female | 197 (47%) | 35 (37%) |
| Induction |  |  |
| IV | 53 (13%) | 6 (6.4%) |
| Inhalation | 365 (87%) | 88 (94%) |
| Airway |  |  |
| LMA | 410 (98%) | 86 (91%) |
| ETT | 8 (1.9%) | 8 (8.5%) |
| Airway Removal | 350 (84%) | 51 (54%) |
| Awake | 68 (16%) | 43 (46%) |
| Deep | 410 (98%) | 86 (91%) |
| Maintenance |  |  |
| Gas | 401 (96%) | 90 (96%) |
| TIVA | 17 (4.1%) | 4 (4.3%) |
| Wheeze | 71 (17%) | 22 (23%) |
| Cough | 72 (17%) | 22 (23%) |
| ColdOrFlu | 68 (16%) | 24 (26%) |
| Unknown | 0 | 1 |
| WheezeExercise | 60 (14%) | 11 (12%) |
| PreviousAsthma | 64 (15%) | 15 (16%) |
| Hayfever | 127 (30%) | 20 (21%) |
| Eczema | 113 (27%) | 17 (18%) |
| Unknown | 0 | 1 |
| AsthmaGT2Family | 140 (33%) | 28 (30%) |
| EczemaGT2Family | 137 (33%) | 20 (21%) |
| HayfeverGT2Family | 197 (47%) | 33 (35%) |
| CaregiverSmoking | 111 (27%) | 24 (26%) |

### 3.3.1 Matching with complete cases

Begin by considering complete cases, reducing the cohort size to 471 (277 receiving clonidine; 84 having PRAE at PACU and/or emergence). Fit a logistic regression model for being in the clonidine group on the mentioned variables, and match.

There is overlap between the estimated propensity scores for the treatment and control groups (Figure 5).

**Figure 5** Density plot of propensity scores calculated for participants who did or did not receive clonidine.

59% of Clonidine cases and 84% of no-Clonidine cases (163 in each group) are matched with a caliper of 0.2*SD (Table 7).

**Table 7** Summary of propensity score matching of the complete-case data.

|  | **No Clonidine** | **Clonidine** |
| --- | --- | --- |
| **Caliper = 0.2*SD** | | |
| All | 194 | 277 |
| Matched | 163 | 163 |
| Unmatched | 31 | 114 |

Every variable is balanced based on SMD (Figure 6) and variance ratio (Figure 7) following matching.

**Figure 6** Mean differences (* indicate standardized mean differences) between covariates in the treatment and control groups. Orange indicated unadjusted (total) sample; blue indicates adjusted (matched) sample.

**Figure 7** Variance ratios between continuous covariates in the treatment and control groups.

**Table 8** Comparison of participant characteristics between clonidine treatment groups for the participants in the matched complete-case dataset. Percentages are shown column-wise.

| **Characteristic** |  | **Clonidine = 0** | **Clonidine = 1** |
| --- | --- | --- | --- |
| n |  | 163 | 163 |
| Trial (%) | BEE | 121 (74.2) | 122 (74.8) |
|  | EMPHASIS | 17 (10.4) | 22 (13.5) |
|  | OSATS2 | 25 (15.3) | 19 (11.7) |
| YearOfSurgery (%) | 2018 | 9 (5.5) | 12 (7.4) |
|  | 2019 | 23 (14.1) | 21 (12.9) |
|  | 2020 | 102 (62.6) | 95 (58.3) |
|  | 2021 | 29 (17.8) | 35 (21.5) |
| Site.cat (%) | Public | 146 (89.6) | 141 (86.5) |
|  | Private | 17 (10.4) | 22 (13.5) |
| Age.f (%) | 0 to 5.9 | 77 (47.2) | 68 (41.7) |
|  | 6 to 8.9 | 58 (35.6) | 63 (38.7) |
|  | 9 and over | 28 (17.2) | 32 (19.6) |
| WeightKG (mean (SD)) |  | 27.74 (14.90) | 28.93 (14.25) |
| HeightCM (mean (SD)) |  | 121.11 (17.76) | 122.44 (19.50) |
| BMI (mean (SD)) |  | 17.86 (4.29) | 18.25 (3.89) |
| ASA (%) | 1 | 62 (38.0) | 66 (40.5) |
|  | 2 | 100 (61.3) | 95 (58.3) |
|  | 3 | 1 (0.6) | 2 (1.2) |
| Sex (%) | Male | 90 (55.2) | 82 (50.3) |
|  | Female | 73 (44.8) | 81 (49.7) |
| Wheeze (%) | YES | 35 (21.5) | 31 (19.0) |
|  | NO | 128 (78.5) | 132 (81.0) |
| Nocturnal Dry Cough (%) | YES | 31 (19.0) | 29 (17.8) |
|  | NO | 132 (81.0) | 134 (82.2) |
| ColdOrFlu (%) | YES | 25 (15.3) | 23 (14.1) |
|  | NO | 138 (84.7) | 140 (85.9) |
| WheezeExercise (%) | YES | 27 (16.6) | 21 (12.9) |
|  | NO | 136 (83.4) | 142 (87.1) |
| PreviousAsthma (%) | YES | 30 (18.4) | 24 (14.7) |
|  | NO | 133 (81.6) | 139 (85.3) |
| Eczema (%) | YES | 42 (25.8) | 41 (25.2) |
|  | NO | 121 (74.2) | 122 (74.8) |
| AsthmaGT2Family (%) | YES | 60 (36.8) | 53 (32.5) |
|  | NO | 103 (63.2) | 110 (67.5) |
| EczemaGT2Family (%) | YES | 52 (31.9) | 46 (28.2) |
|  | NO | 111 (68.1) | 117 (71.8) |
| CaregiverSmoking (%) | YES | 46 (28.2) | 48 (29.4) |
|  | NO | 117 (71.8) | 115 (70.6) |
| Induction (%) | IV | 18 (11.0) | 23 (14.1) |
|  | Inhalation | 145 (89.0) | 140 (85.9) |
| Airway (%) | LMA | 157 (96.3) | 158 (96.9) |
|  | ETT | 6 (3.7) | 5 (3.1) |
| AirwayRemoval (%) | Awake | 124 (76.1) | 130 (79.8) |
|  | Deep | 39 (23.9) | 33 (20.2) |
| Maintenance (%) | Gas | 160 (98.2) | 158 (96.9) |
|  | TIVA | 3 (1.8) | 5 (3.1) |

### 3.3.2 Matching with m = 100 imputations

**Figure 8** Mean differences (*indicate standardized mean differences) between covariates in the treatment and control groups. Orange indicated unadjusted (total) sample; blue indicates adjusted (matched) sample. The range lines around points indicate the range of values across all m = 100 imputations.

**Figure 9** Variance ratios between continuous covariates in the treatment and control groups. The range lines indicate the range of values across all of the m = 100 imputed datasets.

**Table 9** Pooled summary of propensity score matching for the m = 100 imputed datasets.

|  | **No Clonidine** | **Clonidine** |
| --- | --- | --- |
| **caliper = 0.2*SD** | | |
| All | 203.6 | 308.4 |
| Matched | 173.7 | 173.7 |
| Unmatched | 29.9 | 134.8 |

## 3.4 Analysis of matched datasets

### 3.4.1 PRAE at Emergence and/or PACU

From the matched complete-case dataset, 22.1% of participants who were not treated with clonidine had any PRAE at emergence and/or PACU, compared with 17.2% of patients who did receive clonidine pre- or intraoperatively. Despite the small apparent difference in PRAE rates between groups, single-variable logistic regression models on the multiply imputed and matched data (Table 10) showed that there was no statistically significant effect of clonidine on the rate of PRAE at emergence and/or PACU.

**Table 10** Logistic regression models for any PRAE at emergence and/or PACU on treatment with clonidine, using multiply imputed and complete-case datasets following matching.

| **m** | **term** | **estimate** | **p.value** | **2.50%** | **97.50%** | **OR (95% CI)** |
| --- | --- | --- | --- | --- | --- | --- |
| 100 | (Intercept) | -1.238 | < 0.001 | -1.607 | -0.869 | 0.29 (0.2, 0.419) |
|  | Clonidine = 1 (REF = 0) | -0.279 | 0.334 | -0.846 | 0.289 | 0.757 (0.429, 1.335) |
| CCA | (Intercept) | -1.261 | <0.001 | -1.645 | -0.902 | 0.283 (0.193, 0.406) |
|  | Clonidine = 1 (REF = 0) | -0.312 | 0.266 | -0.869 | 0.235 | 0.732 (0.420, 1.265) |

We can also consider a multivariable model, which doubly adjusts for some of the variables which were less balanced in some of the imputed and matched datasets.

**Table 11** Models for any PRAE at PACU and/or Emergence on treatment with clonidine with double-adjustment for some covariates that may have remained unbalanced following propensity score matching.

| **m** | **term** | **estimate** | **p.value** | **2.50%** | **97.50%** | **OR (95% CI)** |
| --- | --- | --- | --- | --- | --- | --- |
| 100 | (Intercept) | 1.826 | 0.692 | -7.238 | 10.89 | 6.209 (0.001, 53637.3) |
|  | Clonidine = 1 (REF = 0) | -0.302 | 0.309 | -0.885 | 0.281 | 0.739 (0.413, 1.324) |
|  | WeightKG | -0.026 | 0.749 | -0.185 | 0.133 | 0.974 (0.831, 1.142) |
|  | HeightCM | -0.021 | 0.569 | -0.095 | 0.052 | 0.979 (0.909, 1.053) |
|  | BMI | 0.01 | 0.939 | -0.255 | 0.276 | 1.01 (0.775, 1.318) |
|  | Sex = Female (REF = Male) | -0.217 | 0.462 | -0.797 | 0.363 | 0.805 (0.451, 1.438) |
| CCA | (Intercept) | 0.062 | 0.898 | -9.292 | 8.520 | 1.064 (0, 5011.964) |
|  | Clonidine = 1 (REF = 0) | -0.294 | 0.308 | -0.865 | 0.269 | 0.745 (0.421, 1.309) |
|  | WeightKG | -0.044 | 0.560 | -0.210 | 0.092 | 0.957 (0.81, 1.097) |
|  | HeightCM | -0.011 | 0.770 | -0.077 | 0.066 | 0.989 (0.925, 1.068) |
|  | BMI | 0.062 | 0.630 | -0.186 | 0.330 | 1.064 (0.83, 1.391) |
|  | Sex = Female (REF = Male) | -0.063 | 0.827 | -0.636 | 0.503 | 0.939 (0.53, 1.654) |

**According to this analysis, taking clonidine pre- or intraoperatively has no significant effect on PRAE incidence at emergence and/or PACU**. This result is replicated in the CCA model and in the multiply imputed model.

### 3.4.2 PRAE at Emergence

This analysis considers any PRAE at emergence, regardless of whether participants would go on to have PRAE at PACU. From the matched complete-case dataset, 6.7% of patients in the no-clonidine group had PRAE at emergence, compared with 5.5% of patients who did receive clonidine. This corresponds to only 20 out of 326 patients in the matched CCA dataset having PRAE at emergence. Logistic regression models for PRAE at emergence show that there is no statistically significant difference in incidence between clonidine treatment groups (Table 12).

**Table 12** Logistic regression models for any PRAE at emergence on treatment with clonidine, using multiply imputed and complete-case datasets following matching.

| **m** | **term** | **estimate** | **p.value** | **2.50%** | **97.50%** | **OR (95% CI)** |
| --- | --- | --- | --- | --- | --- | --- |
| 100 | (Intercept) | -2.726 | < 0.001 | -3.375 | -2.077 | 0.065 (0.034, 0.125) |
|  | Clonidine = 1 (REF = 0) | -0.282 | 0.582 | -1.291 | 0.726 | 0.754 (0.275, 2.067) |
| CCA | (Intercept) | -2.626 | < 0.001 | -3.298 | -2.063 | 0.072 (0.037, 0.127) |
|  | Clonidine = 1 (REF = 0) | -0.214 | 0.645 | -1.148 | 0.696 | 0.808 (0.317, 2.006) |

Given the small number of participants having PRAE at emergence, multiple-variable models will not be considered. **We can conclude that treatment with clonidine is not related to probability of experiencing PRAE at emergence.**

### 3.4.3 PRAE at PACU

This analysis considers any PRAE at PACU, regardless of whether participants had PRAE at emergence. From the matched CCA dataset, 18.4% of patients who did not receive clonidine experienced PRAE in PACU, compared with 14.1% of patients who did receive clonidine.

**Table 13** Logistic regression models for any PRAE at PACU on treatment with clonidine, using multiply imputed and complete-case datasets following matching.

| **m** | **term** | **estimate** | **p.value** | **2.50%** | **97.50%** | **OR (95% CI)** |
| --- | --- | --- | --- | --- | --- | --- |
| 100 | (Intercept) | -1.41 | 0 | -1.799 | -1.02 | 0.244 (0.165, 0.361) |
|  | Clonidine = 1 (REF = 0) | -0.279 | 0.354 | -0.871 | 0.313 | 0.757 (0.419, 1.368) |
| CCA | (Intercept) | -1.489 | <0.001 | -1.903 | -1.108 | 0.226 (0.179, 0.330) |
|  | Clonidine = 1 (REF = 0) | -0.317 | 0.295 | -0.918 | 0.273 | 0.728 (0.399, 1.314) |

We can also consider whether having any PRAE at emergence impacted the probability of having PRAE in PACU. Including PRAE at emergence as a binary covariate in the CCA model for PRAE at PACU does not impact the significance of treatment with clonidine, although as expected those who had PRAE at emergence had a higher probability of PRAE in PACU (OR 4.828 95% CI 1.845, 12.374 p = 0.001 for the matched CCA, with similar results for the pooled multiply imputed models).

**We can conclude that treatment with clonidine is not significantly related to the probability of experiencing PRAE at PACU.**

### 3.4.4 PACU Length of stay

We will assume that the factors impacting PACU length of stay are the same as those which effect PRAE incidence, therefore the same imputed and matched datasets can be used as for the outcome of PRAE.

The distribution of PACU length of stay for the matched CCA data is given in Figure 10. Similarly to the unmatched data (shown in Figure 2), there is little apparent difference in mean tendency of the lengths of stay between the two groups (mean (SD) of 48.2 (22.5) for those receiving no clonidine, compared with 50.0 (23.5) for those who did receive clonidine).

**Figure 10** Distribution of PACU lengths of stay for those in the matched CCA (complete case analysis) dataset who did or did not receive clonidine pre- and/or intraoperatively.

Residuals from the linear regression model for PACU LOS were positively skewed (violating the assumption of normally distributed residuals), so the outcome PACU length of stay was log-transformed; the residuals from the models of log(PACU LOS) were normally distributed, therefore the estimates and p-values from the model for the log transform are shown in Table 14.

**Table 14** Linear models for the log of PACU duration on treatment with clonidine pre- or intraoperatively, for multiply imputed and complete-case matched datasets.

| **m** | **term** | **estimate** | **p.value** | **2.50%** | **97.50%** |
| --- | --- | --- | --- | --- | --- |
| 100 | (Intercept) | 3.787 | < 0.001 | 3.718 | 3.856 |
|  | Clonidine = 1 (REF = 0) | 0.025 | 0.601 | -0.07 | 0.121 |
| CCA | (Intercept) | 3.787 | < 0.001 | 3.726 | 3.851 |
|  | Clonidine = 1 (REF = 0) | 0.036 | 0.434 | -0.054 | 0.126 |

Table 15 shows models for the log of PACU LOS on treatment with clonidine considering double-adjusting for some variables which may have residual differences.

**Table 15** Linear models for log of PACU duration (minutes) considering treatment with clonidine and some other possible covariates.

| **m** | **term** | **estimate** | **p.value** | **2.50%** | **97.50%** |
| --- | --- | --- | --- | --- | --- |
| 100 | (Intercept) | 4.504 | < 0.001 | 3.236 | 5.772 |
|  | Clonidine = 1 (REF = 0) | 0.027 | 0.574 | -0.068 | 0.122 |
|  | WeightKG | 0.011 | 0.236 | -0.007 | 0.03 |
|  | HeightCM | -0.006 | 0.253 | -0.015 | 0.004 |
|  | BMI | -0.018 | 0.336 | -0.055 | 0.019 |
|  | Sex = Female (REF = Male) | -0.053 | 0.280 | -0.149 | 0.043 |
| CCA | (Intercept) | 4.981 | < 0.001 | 3.728 | 6.233 |
|  | Clonidine = 1 (REF = 0) | 0.041 | 0.373 | -0.049 | 0.132 |
|  | WeightKG | 0.018 | 0.057 | -0.000 | 0.037 |
|  | HeightCM | -0.010 | 0.050 | -0.019 | -0.000 |
|  | BMI | -0.029 | 0.126 | -0.065 | 0.008 |
|  | Sex = Female (REF = Male) | -0.051 | 0.275 | -0.142 | 0.040 |

## 3.5 Multivariable model on Raw Cohort

Consider modelling PRAE at Emergence and/or PACU for the original, not imputed, not matched dataset. In this model, we will adjust for differences between the participants who were and were not given clonidine by including covariates in the model for PRAE.

Considering a CCA of the unmatched data for the outcome of PRAE at Emergence/PACU with all variables which were used in the matching algorithm as covariates, model estimates are given in Table 16, along with the reduced model following backwards stepwise variable reduction based on AIC. Considering interaction terms does not alter the final model nor the significance level of the effect of Clonidine on postoperative PRAE.

**Table 16** Multivariable models for postoperative PRAE considering the raw, complete case dataset.

|  |  | **Before stepwise selection** | | | **After stepwise selection** | | |
| --- | --- | --- | --- | --- | --- | --- | --- |
| **Variable** | **Level** | **OR (95% CI)** | **PValue** | **Sig.** | **OR (95% CI)** | **PValue** | **Sig.** |
| Intercept |  | 0.009 (0, 74.739) | 0.322 |  | 0.118 (0.027, 0.537) | 0.005 | ** |
| Clonidine (REF = NO) | Yes | 0.676 (0.383, 1.189) | 0.174 |  | 0.616 (0.361, 1.047) | 0.074 |  |
| Trial (REF = BEE) | EMPHASIS | 1.656 (0.548, 4.536) | 0.344 |  | 1.704 (0.676, 3.916) | 0.229 |  |
|  | OSATS2 | 1.756 (0.515, 6.141) | 0.369 |  | 2.063 (0.987, 4.297) | 0.053 |  |
| Year of surgery (REF = 2018) | 2019 | 0.81 (0.205, 3.086) | 0.759 |  |  |  |  |
|  | 2020 | 0.654 (0.161, 2.675) | 0.549 |  |  |  |  |
|  | 2021 | 0.376 (0.084, 1.665) | 0.195 |  |  |  |  |
| Site (REF = Public) | Private | 0.217 (0.068, 0.604) | 0.006 | ** | 0.24 (0.088, 0.573) | 0.003 | ** |
| Age, years (REF = 0-5.9) | 6 to 8.9 | 0.496 (0.204, 1.188) | 0.118 |  |  |  |  |
|  | 9 and older | 0.407 (0.072, 1.952) | 0.281 |  |  |  |  |
| Weight (kg) |  | 0.93 (0.794, 1.068) | 0.336 |  | 0.941 (0.906, 0.975) | 0.001 | ** |
| Height (cm) |  | 1.027 (0.955, 1.112) | 0.488 |  |  |  |  |
| BMI (kg m^-2^) |  | 1.173 (0.917, 1.52) | 0.213 |  | 1.131 (1.01, 1.261) | 0.029 | * |
| ASA (REF = I) | II | 0.97 (0.543, 1.744) | 0.919 |  |  |  |  |
|  | III | 1.662 (0.076, 14.644) | 0.678 |  |  |  |  |
| Sex (REF = Male) | Female | 0.828 (0.468, 1.454) | 0.511 |  |  |  |  |
| Wheeze (REF = No) | Yes | 1.404 (0.643, 3.004) | 0.386 |  |  |  |  |
| Nocturnal Cough (REF = No) | Yes | 1.113 (0.55, 2.197) | 0.76 |  |  |  |  |
| Cold or Flu (REF = No) | Yes | 2.104 (1.042, 4.195) | 0.036 | * | 2.006 (1.054, 3.755) | 0.031 | * |
| Wheeze at exercise (REF = No) | Yes | 0.486 (0.181, 1.226) | 0.138 |  |  |  |  |
| Previous asthma (REF = No) | Yes | 1.803 (0.8, 3.975) | 0.147 |  |  |  |  |
| Eczema (REF = No) | Yes | 0.532 (0.255, 1.053) | 0.079 |  | 0.524 (0.265, 0.983) | 0.052 |  |
| AsthmaGT2Family (REF = No) | Yes | 1.05 (0.532, 2.036) | 0.887 |  |  |  |  |
| EczemaGT2Family (REF = No) | Yes | 0.626 (0.29, 1.301) | 0.219 |  |  |  |  |
| Caregiver smoking (REF = No) | Yes | 0.788 (0.412, 1.467) | 0.459 |  |  |  |  |
| Induction (REF = IV) | Inhalation | 1.091 (0.392, 3.408) | 0.873 |  |  |  |  |
| Airway (REF = LMA) | ETT | 2.334 (0.621, 8.662) | 0.202 |  |  |  |  |
| Airway Removal (REF = Awake) | Deep | 2.153 (1.09, 4.187) | 0.025 | * | 2.475 (1.311, 4.614) | 0.005 | ** |
| Maintenance (REF = Gas) | TIVA | 1.716 (0.389, 6.209) | 0.435 |  |  |  |  |

# 4 References

| 1. | R Core Team, *R: A language and environment for statistical computing.,* Vienna: R Foundation for Statistical Computing, 2022. |
| --- | --- |
| 2. | S. v. Buuren and K. Groothuis-Oudshoorn, “mice: Multiple Imputation by Chained Equations in R,” *Journal of Statistical Software,* vol. 45, no. 3, December 2011. |
| 3. | S. van Buuren, Flexible Imputation of Missing Data, 2 ed., CRC Press, 2018. |
| 4. | M. A. Brookhart, S. Schneeweiss, K. J. Rothman, R. J. Glynn and Jerry, “Variable selection for propensity score models,” *Practice of Epidemiology,* 2006. |
| 5. | D. B. Rubin and N. Thomas, “Matching using estimated propensity scores: Relating theory to practice,” *Biometrics,* 1996. |
| 6. | Q.-Y. Zhao, J.-C. Luo, Y. Su, Y.-J. Zhang, G.-W. Tu and Z. Luo, “Propensity score matching with R: Conventional methods and new features,” *Annals of Translational Medicine,* 2021. |
| 7. | F. Pishgar, N. Greifer, C. Leyrat and E. Stuart, “MatchThem:: Matching and weighting after multiple imputation,” *The R Journal,* 2021. |
| 8. | P. C. Austin, D. Giardiello and S. v. Buuren, “Impute-then-exclude versus exclude-then-impute: Lessons when imputing a variable used both in cohort creation and as an independent variable in the analysis model,” *Statistics in Medicine,* vol. 42, no. 10, 2023. |
| 9. | B. S. v. Ungern-Sternberg, K. Boda, N. A. Chambers, C. Rebmann, C. Johnson, P. D. Sly and W. Habre, “Risk assessment for respiratory complications in paediatric anaesthesia: a prospective cohort study,” *Lancet,* 2010. |
| 10. | P. R. Rosenbaum and D. B. Rubin, “The central role of the propensity score in observational studies for causal effects,” *Biometrika,* 1983. |
| 11. | X. I. Yao, X. Wang, P. J. Speicher, E. S. Hwang, P. Cheng, D. H. Harpole, M. F. Berry, D. Schrag and H. H. Pang, “Reporting and Guidelines in Propensity Score Analysis: A systematic review of cancer studies and cancer surgical studies,” *JNCI Natl Cancer Inst,* 2017. |
| 12. | J. A. Reiffel, “Propensity score matching: The devil is in the details, where more may be hidden than you know,” *The American Journal of Medicine,* 2020. |
| 13. | D. Lascano, A. K. Zamora, E. Mahdi, S. Ourshalimian, C. J. Russel, E. Kim and L. I. Kelley-Quon, “Gabapentin is associated with decreased postoperative opioid use and length of stay after appendectomy in children with perforated appendicitis: A PSM Analysis,” *Journal of Pediatric Surgery,* 2023. |
| 14. | D. Bottigliengo, I. Baldi, C. Lanera, G. Lorenzoni, J. Bejko, T. Bottio, V. Tarzia, M. Carrozzini, G. Gerosa, P. Berchialla and D. Gregori, “Oversampling and replacement strategies in propensity score matching: A critical review focussed on small sample size in clinical settings,” *BMC Medical Research Methodology,* 2021. |
| 15. | H. Cham and S. G. West, “Propensity score analysis with missing data,” *Psychological Methods,* vol. 21, no. 3, pp. 427-455, March 2016. |
